# A Mouse Model for Studying Post-Acute Arthritis of Chikungunya

**DOI:** 10.3390/microorganisms9091998

**Published:** 2021-09-21

**Authors:** Aileen Y. Chang, Sarah R. Tritsch, Abigail J. Porzucek, Arnold M. Schwartz, Margaux Seyler-Schmidt, Arielle Glass, Patricia S. Latham, St. Patrick Reid, Gary L. Simon, Christopher N. Mores

**Affiliations:** 1 Department of Medicine, School of Medicine and Health Sciences, George Washington University, 2150 Pennsylvania Ave 5-416, Washington, DC 20037, USA; gsimon@mfa.gwu.edu; 2 Department of Global Health, Milken Institute School of Public Health, George Washington University, Washington, DC 20052, USA; sarahtritsch@email.gwu.edu (S.R.T.); abbporz@gmail.com (A.J.P.); mdeja17@gwmail.gwu.edu (M.S.-S.); arielleglass@email.gwu.edu (A.G.); cmores@email.gwu.edu (C.N.M.); 3 Department of Pathology, School of Medicine and Health Sciences, George Washington University, Washington, DC 20037, USA; arnies@gwu.edu (A.M.S.); pslath@gwu.edu (P.S.L.); 4 Department of Pathology and Microbiology, University of Nebraska Medical Center, Omaha, NE 68182, USA; patrick.reid@unmc.edu

**Keywords:** chikungunya, mouse model, arthritis, arthritis therapy, myositis, bone erosion, synovitis

## Abstract

Chikungunya virus (CHIKV) was introduced to the Americas in 2013, causing two million infections across over thirty countries. CHIKV causes a chronic debilitating arthritis in one fourth of infected individuals and currently evidence-based targeted therapies for the treatment of CHIKV arthritis are lacking. Multiple mouse models of chikungunya have been developed to study acute CHIKV infection. In humans, post-CHIKV arthritis may persist for months to years after viremia from a CHIKV infection has resolved. Therefore, the development of a mouse model of post-acute arthritis of chikungunya may facilitate the study of potential novel therapeutics for this arthritis. In this article we describe the development of a wild-type immunocompetent C57BL/6 mouse model for post-acute arthritis of chikungunya, including a histologic inflammation scoring system, as well as suggestions for how this mouse model may be used to examine the efficacy of novel therapies for CHIKV arthritis.

## 1. Introduction

Chikungunya virus (CHIKV) is an alphavirus spread by mosquitos that was introduced to the Americas in 2013, causing two million infections across over thirty countries [1]. It affects an estimated 1 million people annually [2] and causes persistent arthritis in one fourth of patients that may last for months to years [3] that is responsible for significant morbidity [4] and loss of economic productivity [5]. The arthritic potential of CHIKV is not unique; other alphaviruses such as Mayaro, Sinbis, Ross River, and O’nyong’nyong also cause severe arthritis [6]. There is currently no standard evidence-based treatment for alphavirus-induced arthritis. Investigation of the targeted therapies for post-acute arthritis of chikungunya is needed.

Mouse models of CHIKV infection provide valuable information on the pathogenesis of arthritis [7,8,9,10] and its response to various therapies [11,12,13]. Mouse models demonstrate infiltration of monocytes [8,10], macrophages [8,10], and lymphocytes [9,10] in inflamed joints with significant effects on resident fibroblasts [7,14]. Mice infected with CHIKV demonstrate foot swelling and histologic evidence of acute and persistent arthritis [8,9,10].

Specifically, subcutaneous footpad inoculation of adult wild-type immunocompetent C57BL/6 mice with pathogenic strains of CHIKV results in acute foot swelling, myositis, tenosynovitis, and arthritis [8,9,10,11,12,13]. In this model, while the swelling usually resolves within the first 2 weeks of infection [8,9], chronic disease can be observed histologically for at least 21 days after inoculation [9,10] with skeletal muscle necrosis and inflammation, periosteal bone proliferation, synovitis, synovial hypertrophy, and synovial fibrosis [9].

This wild-type immunocompetent C57BL/6 mouse model has been used to evaluate responses to therapies during the acute CHIKV viremic period via drug administration at 3 days post-inoculation [11,12,13]. Lee et al. further demonstrated the importance of drug administration timing in regards to infection timing by showing that pretreatment with an IL-2 antibody complex prior to CHIKV infection prevented CHIKV arthritis [12], whereas administration of the same IL-2 antibody complex during acute CHIKV infection exacerbated the pro-inflammatory response [13]. In clinical practice, there is a need to identify treatments for the chronic arthritis phase that occurs after CHIKV infection as this is when the majority of patients present for therapy. Therefore, we report the methods and key considerations in the development of a wild-type immunocompetent C57BL/6 mouse model with suggestions for how it can be utilized to study drug therapies for post-acute arthritis of chikungunya and in histologic inflammation scoring systems for outcome measurement.

## 2. Materials and Methods

### 2.1. Ethics Statement

The housing of animals utilized in this study and the methods employed during animal experimental procedures were reviewed and approved by the George Washington University (GWU) Institutional Animal Care and Use Committee (IACUC), which complies with the guidelines stated in the National Institutes of Health’s (NIH) Guide for the Care and Use of Laboratory Animals. These experiments were conducted under the approved GWU IACUC protocol #A456.

### 2.2. Biosafety

All studies with viable CHIKV were performed in certified BSL-3 laboratories in biological safety cabinets and were conducted under the approved GWU Institutional Biosafety Committee protocol #IBC-19-026.

### 2.3. Virus

The chikungunya virus strain used in this study was an ECSA lineage, originally isolated from a human case in South Africa (SAH-2123), received from the World Reference Center for Emerging Viruses and Arboviruses (Galveston, TX, USA). Our use of CHIKV strain SAH2123 from the East, Central, and South African (ECSA) lineage was based on the reported pathogenicity for this lineage as compared with others [15], i.e., the effectiveness in a hamster model of chikungunya pathogenesis [16] and the interesting epidemiologic finding of a lineage replacement (Asian- to ECSA-) event that occurred in Boa Vista, Brazil in 2017 [17].

Briefly, following a single passage in Vero cells (CCL-81, ATCC ex. C. aethiops kidney) in DMEM containing antibiotics and antimycotics, serial ten-fold dilutions of the virus stock were inoculated into six-well plates seeded, similarly, with Vero cells, as described previously [18], or were tested by a quantitative reverse transcription—polymerase chain reaction (qRT-PCR), also as described previously [19]. Viral plaques were visualized with crystal violet and titers were expressed as log10 plaque forming units per milliliter (PFU/mL). The calculated titer of the stock virus prepared above was 1.1E7 PFU/mL, which yielded a predicted limit of detection for the qRT-PCR assay of 0.01 PFU/mL

### 2.4. Animal Studies

C57BL/6J mice (≥8 weeks of age; equal distribution of males and females) were obtained from a distributor (The Jackson Laboratory, https://www.jax.org/ (accessed on 14 September 2021)) and inoculated at 11 weeks of age. Mice were housed in groups of five in micro-isolator cages in the ABSL3 laboratory at GWU. Mice were provided water and a standard commercial mouse food ad libitum throughout the experiments.

Prior to infection, mice were initially anesthetized with isoflurane. Subsequently, mice were subcutaneously injected in the caudoventral aspect of the left hind foot, near the tarsal joint, with 2.75 × 10^5^ PFU of virus, selected based on the 10^5^ PFU Bosco-Lauth et al. [16] inoculum of SAH2123 that resulted 4 days of viremia. Age and sex-matched negative control mice were injected with a similar volume (25 μL) of sterile PBS.

Mice were then euthanized at various timepoints throughout the disease course (7, 15, 21 and 25 days post-infection [DPI]). During experiments, animals were monitored a minimum of once daily for clinical signs associated with illness, including lethargy, abnormal posture, joint swelling, lameness, and limb gnawing. Mice were euthanized via CO_2_ asphyxiation followed by cervical dislocation. All experiments were performed in accordance with GWU IACUC guidelines (protocol #A456).

### 2.5. Viremia Measurement

Blood (200 μL) was collected from the submandibular vein for serum analyses at 48 h post-inoculation to confirm infection and at 8, 10, 12, 14 and 16 DPI to measure viremia. Viral RNA was extracted using the Zymo Research Quick DNA/RNA Kit following the manufacturer’s protocol. Quantification of viral load was done with Invitrogen Superscript III Platinum One Step qRT-PCR Kit. RT-PCR (Invitrogen) was analyzed using a LightCycler 96 Instrument (Roche Diagnostics) with thermal cycling conditions as follows: one cycle at 50 °C for 900 s, one cycle of 95 °C for 120 s, and 45 cycles of 95 °C for 3 s, followed by 55 °C for 30 s and a cooling cycle of 37 °C for 30 s.

### 2.6. Tarsal Joint Measurement

Tarsal joint measurements were performed daily using calipers and were recorded, in mm, by animal identification number by day. Tarsal joint swelling was measured by daily measurement of the tarsal joint using calipers and averaging three measurements. The tarsal joint was measured directly posterior to the tarsal joint foot pad projection on the mouse foot, with only enough pressure to cause slightly flaring of the toes. The degree of inflammation was expressed as the increase relative to the pre-infection measurement (day 0), obtained by the following formula: [(x-day 0)/day 0)], where x is the footpad size measurement for a given day post infection.

### 2.7. Histology

Immediately following euthanasia, hindlimbs were dissected with skin removed and placed directly in 10% formaldehyde for fixation for a minimum of 48 h at 4 °C.

Bones were decalcified in an EDTA solution prepared by dissolving 41.4 g EDTA, 4.4 g sodium hydroxide, 50 g PVP in 1000 mL dH2O over very low heat with a magnetic stir bar until the solution was clear, taking up to 8 h. The solution was cooled to room temperature and the pH was adjusted to 7.0–7.4. After fixation, the tissue was washed in water, and placed in the EDTA solution at 4 °C with agitation changing the decalcification solution every other day to maintain pH between 7.0–7.4, for 28 days.

After decalcification, the tissue was rinsed to remove salt before processing in 1× PBS three times for 5 min each, distilled water three times for 5 min each, 50% ethanol once for 5 min, and 70% ethanol once for 5 min, and stored in 70% ethanol at 4 °C until processing.

Samples were then routinely processed for histopathologic evaluation. Briefly, samples were dehydrated in increasing concentrations of ethanol and embedded in paraffin wax. Subsequently, 5 μm-thick sections were adhered to positively charged glass slides. Sections were routinely stained with hematoxylin and eosin (H&E) for evaluation. Standard Masson’s trichrome stains were performed on select slides. All histologic evaluations were performed by a board-certified pathologist blinded to DPI and inoculation status. All slides were evaluated using an Olympus BK46 microscope and digital images were obtained using a Spot Imaging digital camera and Spot Pathsuite 2.0.

### 2.8. Histologic Inflammation Scoring System

A histologic inflammation scoring system was developed in order to have a semi-quantitative measure that can be used to compare outcomes of pathology in therapeutic studies. Based on a previous study [9], our scoring system focused on the following tissue categories: joint space, including articular cartilage and synovium; skeletal muscle and soft tissue, including tendons and fascia; periosteum; and cortical bone. A microscopic evaluation of bone and joint tissue assessed inflammatory reaction and injury in (1) the synovium, (2) articular cartilage, (3) skeletal muscle and soft tissue, (4) periosteum, and (5) cortical bone. The scoring for each component ranged from zero to two, with zero indicating no injury or inflammation, two representing significant injury and inflammation and one representing intermediate involvement with respect to inflammation or injury. Histologic evaluation assigned a maximum value of two points for muscle and soft tissue injury and inflammation, two points for synovitis, two points for articular cartilage erosion, two points for bone and periosteal reaction, and two points for cortical bone erosions for a total range in score of 0–10 per mouse. The scoring system was based on the maximal degree of inflammation and injury seen at day 7.

## 3. Results

### 3.1. CHIKV SAH2123 Reliably Causes Infection in Adult C57BL/6J Mice at 48 h Post Infection

**Figure 1:**
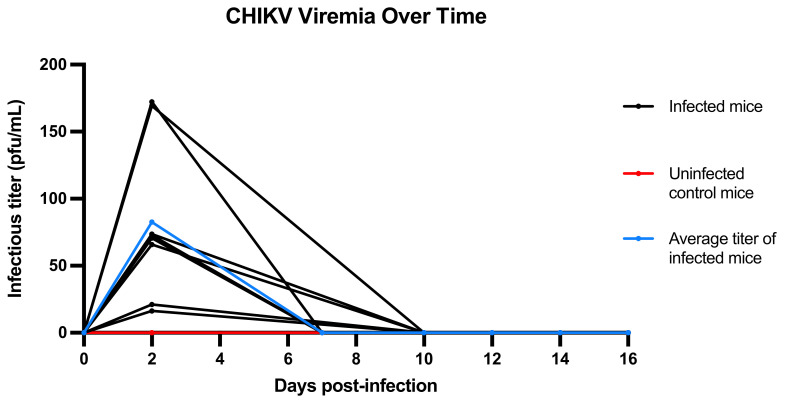
Chikungunya viremia in PFU/mL per mouse by days post infection (DPI). This graphical representation shows viremia in PFU/mL per infected mouse (black lines) and per uninfected control mice (red lines), as well as the average curve for infected mice (blue lines).

All mice inoculated with SAH2123 (*n* = 8) demonstrated significant viremia 48 h post infection, with infectious titers ranging from 16 to 172 PFU/mL (Figure 1). In most infected mice, viremia cleared by 7–10 days, however, very low-level viremia was detectable for some to day 14. Furthermore, one uninfected control mouse, housed with other infected mice, was found to have a very low-level viremia (0.02 PFU/mL) at 48 h post-infection. Other than tarsal joint swelling, the infected mice did not demonstrate overt clinical signs of infection or poor eating.

### 3.2. Viremia Resolves by 10–16 DPI

All mice were negative by RT-PCR for CHIKV by 14 DPI, except for a single mouse, who was negative at 16 DPI Tables S1–S5.

### 3.3. CHIKV SAH2123 Inoculation Results in Ipsilateral Tarsal Joint Swelling for 10 Days

**Figure 2:**
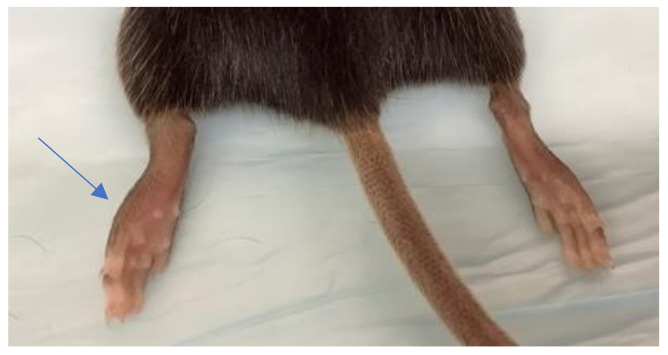
Foot swelling in inoculated foot (arrow) 7 days post inoculation. Note swelling appreciated in the left foot without swelling in the non-inoculated right foot.

**Figure 3:**
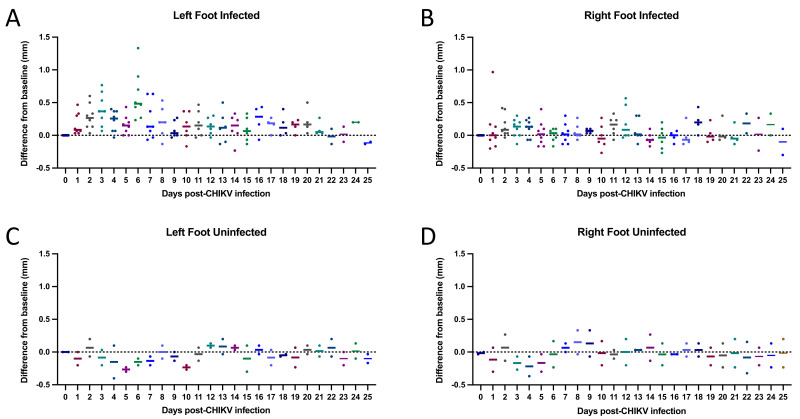
Tarsal joint measurements per mouse by days post infection (DPI). Scatter plots showing the differences from baseline tarsal joint measurements for each day post-CHIKV infection. Mice were injected with approximately 25 μL intradermal of CHIKV inoculum (**A**,**B**) or PBS (**C**,**D**) in the left footpad on Day 0. Tarsal joint measurements were baselined by subtracting Day 0 pre-infection measurements for each mouse from the post-infection measurements. Horizontal solid lines represent the mean difference from baseline for each DPI.

All mice inoculated with SAH2123 developed intense left foot swelling (Figure 2). Tarsal joint measurements per mouse over time are shown in Figure 3. The swelling began one day after inoculation. Daily tarsal joint measurements demonstrate a peak in tarsal joint swelling at 6 DPI. By 10 DPI the tarsal joint swelling had returned to the baseline tarsal joint measure in the left inoculated foot. The right non-inoculated foot did not demonstrate significant foot swelling Tables S1–S5.

### 3.4. Histologic Findings: CHIKV SAH2123 Infection Results in Histologic Evidence of Arthritis, Synovitis, Periostitis and Myositis That Persist to 21 DPI

Pathologic evaluation of direct viral infection of the lower extremity shows a variety of histopathologic features. The areas evaluated in the distal extremity evaluated included skeletal muscle and soft tissue including tendon and fascia, diarthrodial articular components, including synovium and articular cartilage, and bone and periosteum. Minimal inflammation of the non-inoculated extremity relative to the inoculated extremity was observed. Similarly, there was minimal disease in the proximal leg relative to the foot where the inoculation occurred.

**Figure 4:**
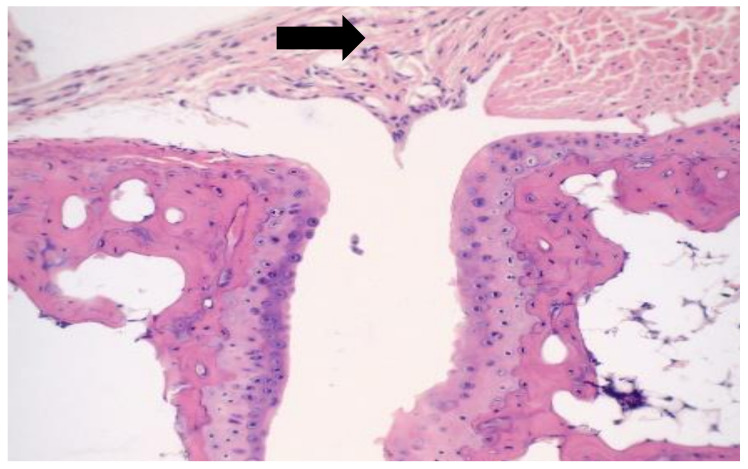
Non-infected mouse with normal joint. Photomicrograph of a normal joint with opposing normal articular cartilage with subchondral bone. There is no evidence of inflammation or erosion. A small amount of synovium (shown with arrow) is present, which appears normal. The joint capsule shows normal connective tissue.

**Figure 5:**
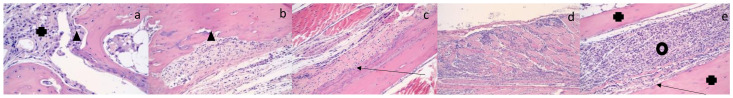
Histologic Evidence of Inflammation by 7 days post-infection (DPI) (**a**–**e**). (**a**) The section shows a lympho-histiocytic synovitis (denoted by a cross) with articular cartilage scalloping and articular cartilage erosion (denoted by a triangle) with extension to cortical bone erosion. The lympho-histiocytic inflammatory response overlies the articular cartilage and cortical bone. There are no neutrophils, no vasculitis and no fibrinoid necrosis. (**b**) The section shows an active periostitis with cortical bone erosion (denoted with a triangle) consisting of inflammatory mononuclear cells and osteoclastic giant cells. There is no evidence of neutrophilic infiltration. Adjacent to the cortical bone erosion is an active mononuclear periostitis. (**c**) Section shows cortical bone with active periostitis, cortical bone erosions and reparative new bone formation (thin arrow). (**d**) Sections demonstrate a mononuclear lympho-histiocytic active myositis with myofiber degeneration and minimal regenerative activity; there is no evidence of healing or fibrosis. (**e**) The section shows interosseous inflammation (denoted with a circle) of soft tissue that is predominantly mononuclear without evidence of granuloma or suppuration. Bone is denoted with a cross. A periostitis is present on cortical bone surface (thin arrow).

**Figure 6:**
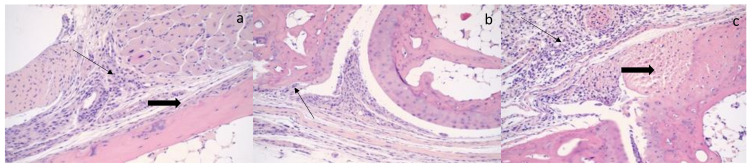
Histologic evidence of inflammation by 21 days post-infection (DPI) (**a**–**c**). (**a**) The histopathology reveals an active myositis with myofiber degenerative change. The lympho-histiocytic inflammatory infiltrate permeates the skeletal muscle (thin arrow) and extends to cortical bone, causing a periostitis with mild cortical bone erosion (thick arrow). (**b**) Section of joint active arthritis with an erosive lympho-histiocytic synovitis with articular cartilage scalloping and articular cartilage erosion with extension to cortical bone erosion (thin arrow). There is joint capsule inflammation, which extends into soft tissue. (**c**) Section of joint arthritis demonstrates an active lympho-histiocytic synovitis (thin arrow) with cartilage damage and erosion. The active joint inflammation shows extensive involvement and erosion of articular cartilage (thick arrow) with inflammatory extension of the joint capsule.

The viral infection resulted in an inflammatory myositis with muscle fiber degeneration, joint and cartilage injury with synovitis, and articulate cartilage erosion and adjacent periostitis with cortical bone erosion. The pathology demonstrated a dominant pattern of inflammatory arthritis with articular cartilage erosion with some adjacent periostitis, cortical bone erosion, and myositis. The arthritis, synovitis, and cartilage injury precede the periostitis and cortical bone erosion, which precedes the inflammatory myositis. The inflammatory infiltrate was predominantly mononuclear, with a lympho-histiocytic infiltrates, scattered plasma cells, and few neutrophils. Digital images of characteristic histologic findings are shown in Figure 4, Figure 5 and Figure 6.

A representative sample of the normal non-infected histology is shown in Figure 4. The normal diarthrodial joint displays opposing normal articular cartilage with subchondral bone without evidence of inflammation or erosion, a small amount of normal synovium, and normal connective tissue within the joint capsule.

At 7 DPI (Figure 5), the histologic sections identify a destructive mononuclear lympho-histiocytic arthritis and periostitis. There is prominent synovitis, articular cartilage and cortical bone erosions, myositis, and periostitis, with no evidence of fibrosis/scarring. The erosive periostitis is associated with cortical bone injury and resorption by inflammatory mononuclear cells and osteoclastic giant cells. Other soft tissue involvement is identified as interosseous inflammation. There is no vasculitis identified and bone marrow show no ischemic necrosis nor suppurative inflammation.

By 21 DPI (Figure 6), histologic evaluation demonstrates persistent evidence of inflammation in the inoculated limb with periostitis with some cortical erosive and new reactive bone formation, myositis with myofiber degenerative change, lympho-histiocytic inflammatory infiltrates, and lympho-histiocytic synovitis, with articular cartilage scalloping. In addition, there is joint capsule inflammation, which extends into soft tissue.

By 25 DPI, one mouse showed continued synovitis and periostitis and myofiber degenerative change, whereas the other mouse showed no evidence of persistent inflammation (synovitis, myositis, tendinitis, or bone or periosteal erosions). At 25 DPI, the mouse with the inflamed extremity had synovitis with cartilage erosion, periostitis with mild cortical bone erosion and mild myositis with degenerative changes and a limited fasciitis.

The uninoculated control mouse, who experienced low level viremia after being housed with infected mice, demonstrated mild inflammation with lymphocytic infiltrates in both feet. The other control mouse was free of any pathologic findings. In the infected mice, the inoculated feet also showed inflammatory changes, however. to a lesser degree than present in the locally infected extremity.

Average histologic inflammation scores over time are shown in Table 1.

In all the infected mice, there was no evidence of granulomas, thrombosis or abscesses. Though articular cartilage and cortical bone erosions were present, there was no evidence of cartilage or bone necrosis, and no evidence of ischemic bone marrow necrosis or osteomyelitis. There was no evidence of soft tissue fat necrosis. There was no evidence of acute vasculitis nor fibrinoid necrosis. No cytopathic viral inclusions were identified.

## 4. Discussion

We present a wild-type immunocompetent C57BL/6 mouse model that can be used to study post-CHIKV arthritis therapies. This model differs from other models in that, in this model, histologic scoring is described to assess joint and muscle inflammation after the mouse is no longer viremic to mirror post-acute arthritis, as opposed to arthritis found during acute infection. Our key findings include that (1) all inoculated mice demonstrated viremia at 48 h post-infection, suggesting that a single 48-h post-inoculation blood draw for viremia assessment would be sufficient to confirm successful infection prior to investigational drug therapy as part of an experimental protocol; (2) there is a window, from 16–21 DPI, wherein the mice are no longer viremic but still display prominent histologic evidence of inflammation, that would be the ideal window for studies of investigational drugs; (3) the composite histologic inflammation score may be of utility for determining efficacy outcomes; and (4) tarsal joint swelling is only present for a limited duration during viremia, which excludes tarsal joint swelling as a valuable endpoint for post-acute arthritis of chikungunya drug studies.

Our findings are similar to the findings of other studies, including the early presence of viremia in adult immunocompetent mice after inoculation with multiple different strains of CHIKV [8,9,10,20] and peak tarsal joint swelling at 6–8 DPI, with the resolution of tarsal swelling by 14 DPI [9] with preservation of the histologic evidence of inflammation to 21 DPI [8,9]. Our finding of a single mouse that was infected via exposure to infected mice suggest that uninfected mice should be housed separately from infected mice. Our histologic findings are similar to that of Goupil et al., demonstrating an active synovitis with articular cartilage erosion, extension to cortical bone with periostitis and cortical bone erosion and an inflammatory myositis with muscle fiber degeneration [9].

Our histopathologic findings differ from Goupil et al. in that we did not find the other features of necrosis described in that report, including necrosis of cartilage or bone, nor evidence of ischemic bone marrow necrosis, and we saw no evidence of osteomyelitis. Furthermore, we did not identify bone marrow necrosis of an ischemic etiology or pathogenesis, nor any local or regional vascular pathology that would contribute to vascular compromise. Moreover, the marrow necrosis of an ischemic pathogenesis should demonstrate fat necrosis with multinucleated giant cell response that was not identified in our histologic images. Finally, the neutrophilic infiltrates found in Goupil et al.’s photomicrographs, which may be interpreted as active osteomyelitis, were also not present in our cases.

This is the first study to our knowledge to follow the mice to 25 DPI. We saw resolution of histologic inflammation in some mice at 25 days, indicating that endpoints for drug studies should be assessed prior to 25 DPI.

We also found that tarsal joint swelling was only significant for a duration of approximately 10 days during viremia, which may preclude the use of tarsal joint swelling as a valuable endpoint for post-CHIKV arthritis drug studies. Another study by Lee et al. [12] documented footpad swelling by daily measurement of the height and breadth of the footpad, using calipers, until Day 14. Perhaps this may be a more sensitive measure of inflammation in the lower extremity and may have greater utility as a parameter for measurement.

A limitation of this study includes the limited number of mice utilized; however, the similarity of our findings in terms of severity and duration of disease with those in other studies is reassuring. Another limitation was the use of a histologic inflammation score that is semi-quantitative as opposed to quantitative. A quantitative score could be developed by counting the numbers of inflammatory T-cells identified by immunohistochemistry per high-power field. However, the benefit of the semi-quantitative score used in this study is that it generates a composite score from multiple elements of tissue histology that contribute to the inflammatory pathology. The composite score is based on a specific assessment of the joint pathology resulting from CHIKV infection and so may be especially well suited to represent clinical response to disease/therapy. In addition, future validation of this model, with arthritis therapies of various durations of treatment, is needed.

## 5. Conclusions

We presented a wild-type immunocompetent C57BL/6 mouse model for studying post-acute arthritis of chikungunya. Our findings suggest the optimal timeframe for testing novel arthritis therapeutics is at 16–21 DPI, using histologic outcomes. Validation of this model using various arthritis therapies is needed. This may serve as a foundation for future studies to develop mouse models for studying chronic post-infection arthritis in other alphaviral infections known to cause arthritis, as well.

## Figures and Tables

**Table 1:** Histologic inflammation score of ipsilateral lower extremity by days post infection (DPI) per mouse. The evaluation consisted of microscopic evaluation of inflammatory reaction and injury of the (1) synovium, (2) articular cartilage, (3) skeletal muscle and soft tissue, (4) periosteum, and (5) cortical bone. The scoring for each component ranged from zero to two, with zero indicating no injury or inflammation, two represents significant injury and inflammation, and one representing intermediate involvement with respect to inflammation or injury for a total range in score of 0–10 per mouse, wherein 10 is the worst pathology.

	7 DPI		15 DPI		21 DPI		25 DPI	
Chikungunya inoculated Mice (*n* = 8)	10	10	5	5	5	4	0	6
Non-inoculated Controls (*n* = 2)							2	0

## Data Availability

The data presented in this study are available in the article or in Supplemental File 1.

## Supplementary Materials

The following are available online at https://www.mdpi.com/article/10.3390/microorganisms9091998/s1, Table S1: PFU, Table S2: Standard Curve, Table S3: Titer Calculation, Table S4: Viremia Chart, Table S5: Tarsal Joint Raw Data.
